# Differing animal welfare conceptions and what they mean for the future of zoos and aquariums, insights from an animal welfare audit

**DOI:** 10.1002/zoo.21677

**Published:** 2022-03-07

**Authors:** Jake S. Veasey

**Affiliations:** ^1^ Care for the Rare c/o, School of Animal Nottingham Trent University Nottingham UK

**Keywords:** affective states, physical health, psychological wellbeing, public opinion, stereotypies, veterinary

## Abstract

Animal welfare is a growing public concern that has the potential to undermine the social license of zoos and aquariums. The lack of consensus on how animal welfare is defined across such a diverse sector combined with and a widespread belief that commercial priorities such as entertaining visitors conflicts with animal welfare, hinders efforts to effectively address this fundamental issue for the sector. Data derived from an audit of habitats across a major North American wildlife attraction revealed that holistic animal welfare assessments undertaken by animal carers embracing three principal constructs of animal welfare, correlated strongly with visitor perceptions of animal happiness. Visitor assessments of animal happiness also correlated with animal carer assessments of social, behavioural and locomotor opportunities and inversely with the prevalence of stereotypic behaviours, supporting the proposition that folk conceptions of animal welfare are more accurate than may have previously been considered to be the case. However, the holistic animal welfare assessment inversely correlated with assessments of a habitat's capacity to safeguard welfare as determined by the facility's veterinary staff, supporting the proposition that tensions exist between physical and psychological components of captive animal welfare provisioning. This further underlines the importance of clarity on how animal welfare is conceived when developing institutional animal welfare strategies. Finally, the data also showed that both holistic animal welfare assessments and visitor perceptions of animal happiness strongly correlated with the level of enjoyment experienced by visitors, challenging the belief that animal welfare competes with the commercial priorities of zoos and aquariums. The audit supports the case that maintaining high animal welfare is a commercial imperative as well as a moral obligation for zoos and aquariums and underlines the necessity to utilize conceptions of animal welfare that acknowledge the centrality of the affective states of animals in maintaining those standards.

## Research highlights

- Most zoo animal welfare stakeholder groups do not have publicly available animal welfare definitions and those that do, differ from the reported scientific consensus. For wildlife attractions, this ambiguity has the potential to create confusion, misalignment of effort, conflict, and an erosion of trust with the general public. An animal welfare audit undertaken across a large wildlife attraction encompassing a range of stakeholder perspectives and welfare conceptions revealed differing alignments; the performance of habitats from a veterinary management perspective inversely correlated with a holistic animal welfare assessment undertaken by animal care staff utilising a suite of broad‐based criteria, encompassing three principle animal welfare constructs relating to natural living, effective states and health/biological functioning. This holistic assessment correlated with visitor perceptions of animal happiness as well as their enjoyment of those habitats. Visitor assessments of welfare also correlated with animal carer assessments of social, behavioural and locomotor opportunities and with the prevalence of stereotypic behaviours. The findings of this audit support the case that tensions can exist between veterinary and holistic animal welfare‐based priorities in achieving peak captive animal welfare and support the case for a consensus in defining animal welfare centred around the affective states of animals. Finally, the audit suggests folk understandings of animal welfare are more accurate than may have been considered to be the case,  and that welfare is important in shaping visitor experiences of wildlife attractions, illustrating the strategic as well as moral importance of animal welfare to wildlife attractions.

## Graphical abstract

A holistic welfare audit of habitats within a large wildlife attraction correlated with visitor perceptions of animal happiness and visitor enjoyment but inversely correlated with a veterinary‐based welfare audit. These findings underline the significance of differing welfare conceptions in establishing institutional animal welfare management priorities and the potential tensions between physical and psychological priorities in captive animal care.

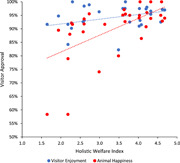

## 1 INTRODUCTION

Despite the increased awareness of and sympathy for the welfare and conservation of wild animals around the world (see Marinova & Fox, 2019; Robbins et al., 2018; Webb et al., 2019), wild animal populations have nonetheless collapsed with one million species now facing extinction (United Nations, 2021). While captive breeding has reportedly played a role more than half of the cases where extinction has been prevented for birds and mammals (Bolam et al., 2020), concerns about the welfare of wild animals in captivity jeopardises the social license of zoos and aquariums, threatening this increasingly important conservation tool (Clubb & Mason, 2003, 2007; Mellor et al., 2018). This poses an ethical dilemma for societies motivated by concerns for captive animal welfare as well as species conservation, since the extent to which captive breeding can support expanding conservation needs, will inevitably be constrained by both legitimate and ill‐founded concerns over the welfare of wild animals in zoos and aquariums (see Aspinall, 2019; Balmford et al., 2011; Born Free, 2021; Clubb & Mason, 2003; 2007; Mellor et al., 2018; PETA, 2021; N. A. Rose & Parsons, 2019).

To safeguard their social license, conservation potential and long‐term commercial viability, it is essential, therefore, that zoos and aquariums identify and energetically address the genuine welfare issues that exist within their institutions, and proactively assuage public concerns over the welfare of animals in their care, while understanding that actual animal welfare issues and perceived animal welfare concerns may not always be perfectly aligned.

Effectively addressing such issues across a sector as taxonomically, culturally, philosophically, and geographically diverse as zoos and aquariums where institutions are routinely judged according to the performance of the least accomplished facilities, will inevitably be a challenge. This is exacerbated by a tendency by many zoo and aquarium professionals to disregard nonexpert opinions on welfare matters as being anthropogenic, subjective, and superficial (see Melfi & Mccormick, 2004; Wolfensohn et al., 2018) and a widespread belief across a range of organisations that animal‐related priorities compete with commercial objectives (see Born Free, 2021; Keulartz, 2015; Morino, 2017; PETA, 2021; N. A. Rose & Parsons, 2019; Wolfensohn et al., 2018). However, arguably the biggest constraint on the systematic advancement in animal welfare provisioning across the sector is the lack of a universal conception of animal welfare (see Fraser, 2008a; Hewson, 2003; Marinova & Fox, 2019; Robbins et al., 2018; Veasey, 2017) and a tendency instead, to establish priorities focused more on how animal welfare is measured rather than how animals experience it (Veasey, 2017).

A review of the websites of 22 stakeholder organisations encompassing animal welfare, 'anti‐zoo', zoo and aquarium organisations and zoo and aquarium accrediting bodies showed that ten provided definitions of animal welfare with a further four providing some commentary on animal welfare beyond it simply being an objective/goal (see Table 1). While the definitions were relatively diverse in form, only four of the 22 stakeholders (18%); Wild Welfare, the World Association of Zoos and Aquariums, the South East Asian Zoo and Aquarium Association and the Universities Federation of Animal Welfare, emphasised hedonistic conceptions of animal welfare centred around the affective states of animals, reflecting the reported consensus of animal welfare scientists (see Robbins et al., 2018; Webb et al., 2019). This apparent disconnect between scientific conceptions of animal welfare and that of animal welfare stakeholders, supports the concerns expressed by Veasey (2017) in relation to how welfare definitions are applied in captive animal welfare management.

**Table 1:** A summary of the range of publicly available definitions provided by representative stakeholders in the welfare of animals within zoos and aquariums

Organization		Welfare definition / comments	Reference
American Humane		No definition identified.	https://www.americanhumane.org
			Accessed 23 April 2021.
American Veterinary Medical Association		“Animal welfare means how an animal is coping with the conditions in which it lives. An animal is in a good state of welfare if (as indicated by scientific evidence) it is healthy, comfortable, well‐nourished, safe, able to express innate behavior, and if it is not suffering from unpleasant states such as pain, fear, and distress. Good animal welfare requires disease prevention and veterinary treatment, appropriate shelter, management, nutrition, humane handling, and humane slaughter. Animal welfare refers to the state of the animal; the treatment that an animal receives is covered by other terms such as animal care, animal husbandry, and humane treatment.”	https://www.avma.org/resources/animal-health-welfare/animal-welfare-what-it
			Accessed 23 April 2021.
Animal Welfare Institute		No definition identified.	https://awionline.org
			Accessed 23 April 2021.
Association of Zoos and Aquariums		“Animal Welfare refers to an animal's collective physical, mental, and emotional states over a period of time, and is measured on a continuum from good to poor.”	https://www.aza.org/animal_welfare_committee?locale=en
			Accessed 23 April 2021.
Born Free Foundation		No definition identified.	https://www.bornfree.org.uk
			Accessed 23 April 2021.
British and Irish Association of Zoos and Aquariums		No definition identified, but listed the following factors to be considered when assessing welfare: Physical health, mental health, social life, enclosure space and environmental enrichment.	https://biaza.org.uk/animal-welfare
			Accessed 23 April 2021.
Canadian Association of Zoos and Aquariums		“CAZA defines Animal Welfare as an animal's physical, mental, and emotional states over a period of time, and is measured on a continuum from good to poor.”	https://caza.ca/wp-content/uploads/2016/06/CAZA-Policy-on-Use-of-Animals-in-Educational-Programming.doc.pdf
			Accessed 23 April 2021.
Center for Zoo and Aquarium Animal Welfare and Ethics		No definition identified, but stated “An individual's overall mental, physical and emotional state (referred to as welfare or well‐being) is determined only by that individual.”	https://czaw.org/about/
			Accessed 23 April 2021.
European Association of Zoos and Aquariums		“Animal welfare refers to the physiological and psychological health of an animal – effectively, this is how the individual animal is coping, both mentally and physically with their circumstances.”	https://www.eaza.net/about-us/areas-of-activity/animal-welfare/
			Accessed 23 April 2021.
People for the Ethical Treatment of Animals		No definition identified, however, the following statement was made. “Animal welfare theories accept that animals have interests but allow these interests to be traded away as long as there are some human benefits that are thought to justify that sacrifice. Animal rights means that animals, like humans, have interests that cannot be sacrificed or traded away just because it might benefit others. However, the rights position does not hold that rights are absolute; an animal's rights, just like those of humans, must be limited, and rights can certainly conflict. Animal rights means that animals are not ours to use for food, clothing, entertainment, or experimentation. Animal welfare allows these uses as long as “humane” guidelines are followed”.	https://www.peta.org/about-peta/faq/what-is-the-difference-between-animal-rights-and-animal-welfare/
			Accessed 23 April 2021.
Performing Animal Welfare Society		No definition identified.	http://www.pawsweb.org/index.html
			Accessed 23 April 2021.
Royal College of Veterinary Surgeons		No definition identified, however, in the course of their strategic plan, the word welfare is used twenty‐one times and every time as part of the phrase “health and welfare”.	https://www.rcvs.org.uk/news-and-views/publications/rcvs-strategic-plan-2020-2024/
			Accessed 23 April 2021.
Royal Society for the Prevention of Cruelty to Animals		No definition identified.	https://www.rspca.org.uk/home
			Accessed 23 April 2021.
South East Asian Zoos Association		“Animal Welfare refers to the psychological state of the animal. The animal's welfare state will be good when it experiences positive sensations that may result when the animal is in good health, and readily express a range of normal and positive behaviors. It involves a human responsibility to provide appropriate housing, veterinary treatment, behavioral management, nutrition, disease management, responsible care and use, humane handling and, when necessary, humane euthanasia.”	http://www.seaza.asia/wp-content/uploads/2020/03/SEAZA-Standard-on-Animal-Welfare-English.pdf
			Accessed 23 April 2021.
Universities Federation for Animal Welfare		“Ensuring good welfare is about more than ensuring good health. Animal welfare is about the quality of animals' lives: their feelings. It is now widely agreed, although it was not always so, that many species are sentient ‐ they have the capacity to feel pain and distress, they can suffer and, conversely, be aware of pleasant feelings ‐ and that this matters morally.”	https://www.ufaw.org.uk/about-ufaw/ufaw-and-animal-welfare
			Accessed 23 April 2021.
Whale Sanctuary Project		No definition identified.	https://whalesanctuaryproject.org/
			Accessed 23 April 2021
Wild Welfare		“Animal welfare science is used to define how an animal is coping with the conditions in which it lives. It considers actual feelings and sensations that an animal experiences and refers to the psychological well‐being of the individual.”	https://wildwelfare.org/animal-welfare/
			Accessed 28 January 2022
World Animal Protection		“Animal welfare concerns the physical and mental well‐being of animals and involves considerations of how animals evolved and their natural environments. It is a description of the state of animals and the effect on them of care or mistreatment.”	https://www.worldanimalprotection.ca/sites/default/files/media/ca_-_en_files/case_for_a_udaw_tcm22-8305.pdf
			Accessed 23 April 2021.
World Association of Zoos and Aquariums		“Animal welfare refers to a state that is specific for every individual animal; it is how the animal experiences its own world and life through its association with pleasant experiences specific for that species such as vitality, affection, safety and excitement, or unpleasant experiences such as pain, hunger, fear, boredom, loneliness and frustration.”	WAZA 2021. WAZA's Approach to Animal Welfare. Accessed 23 April 2021.
			https://www.waza.org/priorities/animal-welfare/our-approach-to-animal-welfare/
			Accessed 23 April 2021.
World Organization for Animal Health		“..animal welfare means ‘the physical and mental state of an animal in relation to the conditions in which it lives and dies. The guiding principles which inform the OIE's (Office International des Epizooties) work on the welfare of terrestrial animals include the ‘Five Freedoms’. Developed in 1965, and widely recognized, the five freedoms describe society's expectations for the conditions animals should experience when under human control, namely: freedom from hunger, malnutrition and thirst; freedom from fear and distress; freedom from heat stress or physical discomfort; freedom from pain, injury and disease; and freedom to express normal patterns of behavior.”	https://www.oie.int/en/animal-welfare/animal-welfare-at-a-glance/
			Accessed 23 April 2021.
Zoo and Aquarium Association (Australasia)		No definition identified, however, ZAA state “The one thing that must underpin everything else we do is positive animal welfare. We don't believe in settling with just “not bad”, we want the animals under our care to experience “great” welfare and live fulfilling lives.” and “ZAA uses the Five Domains Model, developed by Massey University.”	https://www.zooaquarium.org.au/public/Animal-Welfare/Public/Animal-Welfare/Animal-welfare.aspx?hkey=8969dee1-84c9-4652-8e49-cd1fff4b131e
			Accessed 23 April 2021.
Zoo Check (Canada)		No definition identified.	https://www.zoocheck.com
			Accessed 23 April 2021.

Here the significance of differing stakeholder conceptions of animal welfare is evaluated within a single North American wildlife‐based visitor attraction to explore how different conceptions relate to each other, to welfare as it is likely to be experienced by animals, and to the visitor experience as a metric of the commercial offering of zoos and aquariums.

## 2 MATERIALS AND METHODS

A facility‐wide animal welfare audit was undertaken across a large wildlife attraction within North America, principally to guide management and long‐term master‐planning. The scope of the audit and the size of the facility necessitated assessments centred around the performance of habitats rather than the welfare of individual animals which would have been impractical at such a facility that housed many mixed species, populous habitats with a wide range of species including mammals, birds, reptiles, amphibians, fish and invertebrates. The audit comprised three separate assessments undertaken by animal care staff, veterinary staff and visitors, with the latter also providing feedback on their enjoyment of each habitat as a crude proxy for commercial outcomes. All assessments utilized a ranking of one to five with five representing the best possible score and one, the poorest.

The animal carers' assessment was undertaken by staff with at least one year's experience working with the habitat being considered and was based on a broad‐spectrum, holistic animal welfare performance assessment, that considered elements from three principal animal welfare constructs relating to health/biological functioning, natural living and affective states (Fraser, 2008a; Webb et al., 2019; Webb & Robbins, 2019). Staff were inducted into the process and provided with a calibrated ranking guide before undertaking the assessment independently. For habitats with three or more species, animal care and veterinary staff were instructed to assess the species or individuals for which they had the greatest welfare concerns, and for the two habitats in which there were two resident species, staff assessed each species separately. The five criteria used in the animal carers' assessment were:

1. The extent to which resident animals were free to express biologically important motivated behaviors.
2. The extent to which the habitat predisposed resident animals to physical and physiological challenges.
3. The extent to which the social environment was appropriate for the resident animals.
4. The extent to which the resident animals were free to locomote at maximum velocity within the habitat.
5. The extent to which resident animals expressed abnormal or stereotypic behaviors.

Collectively these criteria are referred to as the holistic welfare index (HolisticWI) and reflect an attempt to assess the presence or absence of negative states as well as opportunities to experience positive states through the expression of inherently rewarding, motivated behaviours and cognitive processes, as well as the opportunity to live in biologically appropriate surroundings. As the name implies, the HolisticWI is intended to encapsulate the all‐round welfare requirements of the animals recognizing that their welfare is dependent upon their affective experiences (see Duncan, 2002; Duncan & Petherick, 1991; Mason & Veasey, 2009a, 200b; Veasey, 2017), which in turn is influenced by the opportunities available to them and their physical wellbeing (Veasey, 2017). The HolisticWI is the baseline by which other conceptions are evaluated and is intended to be representative of the welfare of the animal as it experiences it.

To evaluate different welfare conceptions as opposed to different interpretations of a single welfare conception by different stakeholders, the veterinary team at the facility were asked to establish their own criteria for assessing the welfare performance of each habitat. Rather than submitting individual data for each member of the team, the veterinary team chose to undertake their assessment as a group with values provided for each habitat reportedly representing a consensus view of the team. The three criteria established by the veterinary team were:

1. The ease with which emergency care could be provided to animals in need.
2. The ease with which preventative healthcare measures can be provided.
3. The incidence of morbidity and mortality issues within the habitat.

Collectively these criteria are referred to as the veterinary welfare index (VetWI).

The final welfare perspective was derived from visitors who provided scores on a habitat wide basis regardless of the number of species within each habitat and was collected using touch screens adjacent to habitats. The touch screens offered visitors a series of five emojis with which they ranked their opinions on the performance of habitats ranging from a positive response represented by an open‐mouthed smiling face, a neutral response represented by a face that is neither smiling nor sad/angry, through to a negative response represented by a visibly angry face. Following an extensive trial, three mechanisms were put in place to eliminate potentially spurious data from visitors interacting with the touch screens without properly engaging in the content. Firstly, two questions were included relating to each habitat being considered that needed to be answered correctly before the input was included in the analysis. Secondly, if responses to questions were given in a time deemed shorter than it would be possible to read the question and respond, that input was also excluded. Finally, the order in which the emoji‐based ranking was presented from positive to negative was alternately reversed along the horizontal axis, to preclude respondents simply tapping the screen at a specific location and potentially creating a false correlation between separate questions. The questions asked of the visitors in this format were:

1. Are the animals in the habitat healthy?
2. Are the animals in the habitat happy?
3. Did you enjoy viewing the habitat?

Of the three criteria making up the visitor assessment, the first two criteria relating to health and happiness are collectively referred to as the visitor welfare index (VisitorWI). Since visitors to a wildlife attraction are arguably more likely to approve of such facilities in providing appropriate environments for resident animals than those that would actively avoid them, the proportion of visitors approving of habitats was considered as more relevant than an average rank score across the surveys completed. An approval index was therefore calculated based on the percentage of visitors providing a positive ranking; the higher two of the five options available to visitors for each criterion represented by the closed and open‐mouthed smiling emojis.

In all three assessments, the one to five rankings were set out such that the lower the score, the poorer the welfare performance is likely to be; so for example, a rank of one, respectively, represented the categories including the least happy, most stereotypic, least able to locomote, most predisposed to physical/physiological challenges, least able to support the provision of emergency care and so on.

## 3 RESULTS

In total, animal care staff undertook 1220 assessments across 133 habitats yielding an average of 9.2 assessments for each habitat. Visitors undertook a total of 2958 assessments across 29 habitats of which, 1337 assessments (45.2%) passed the validation filters, with each habitat subsequently yielding an average of 46.1 validated visitor assessments. The veterinary team collectively assessed 108 habitats.

**Figure 1:**
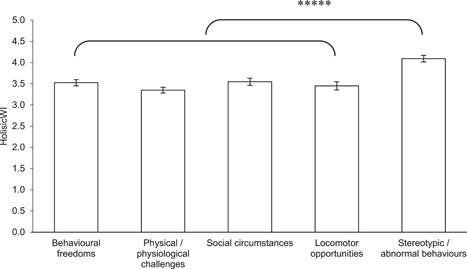
Scores for each of the five criteria making up the HolisticWI. A Friedman rank‐sum test confirmed there to be a statistically significant difference between the metrics (*χ* ^2^ = 170.165 (4, *N* = 133), *p* < .00001) with a Nemenyi post‐hoc test revealing assessments stereotypies and abnormal behaviours were significantly different from all other assessment criteria at the same level of significance (*p* < .00001)

**Figure 2:**
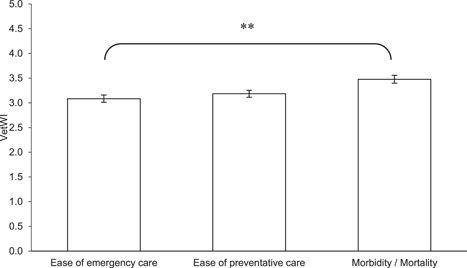
Scores for each of the three criteria making up the VetWI. A Friedman rank‐sum test confirmed there to be a statistically significant difference between the metrics (*χ* ^2^ = 16.182 (2, *N* = 108), *p* < .0005) with a Nemenyi post‐hoc test revealing ease of emergency care was significantly different from morbidity and mortality (*p* < .01)

**Figure 3:**
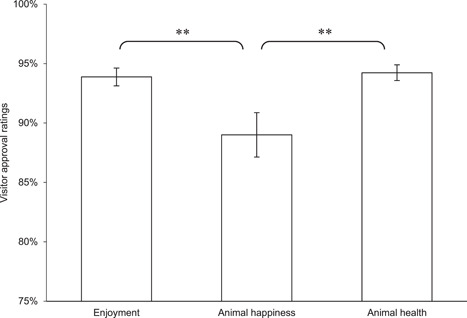
Scores for each of the three criteria making up the visitors' assessments of habitats. A Friedman rank sum test confirmed there to be a statistically significant difference between the metrics (*χ* ^2^ = 10.220 (2, *N* = 29), *p* < .01) with a Nemenyi post‐hoc test revealing assessments of animal happiness were significantly different from assessments of visitor enjoyment and assessments of animal health at the same level of significance (*p* < .05)

To determine whether the criteria making up each of the three stakeholder group welfare assessments were not simply mirroring each other, Friedman rank‐sum tests were carried on each. For the five metrics that make up the HolisticWI; there was found to be a statistically significant difference between the metrics (*χ* ^2^ = 170.165 (4, *N* = 133), *p* < .00001) with a Nemenyi post‐hoc test revealing assessments of stereotypies and abnormal behaviours were significantly different from all other assessment criteria at the same level of significance (*p* < .00001, see Figure 1). The three metrics that make up the VetWI were found to be a statistically significant different (*χ* ^2^ = 16.182 (2, *N* = 108), *p* < .0005) with a Nemenyi post‐hoc test revealing ease of emergency care was significantly different from morbidity and mortality (*p* < .01, see Figure 2). Finally, there was found to be a statistically significant between the three metrics making up the visitor assessments (*χ* ^2^ = 10.220 (2, *N* = 29), *p* < .01) with a Nemenyi post‐hoc test revealing assessments of animal happiness were significantly different from assessments of visitor enjoyment and assessments of animal health at the same level of significance (*p* < .05), see Figure 3).

As animal welfare is a nuanced concept influenced by, and manifest in multiple factors (Dawkins, 2006, 2017; Fraser, 2008a, 2008b; Mason & Mendl, 1993; Veasey, 2017, 2019, 2020a, 2020b; Webb et al., 2019, Webb & Robbins, 2019) and the purpose of this paper was to explore the relationship between differing multifaceted animal welfare stakeholder conceptions, analyses included the consolidated scores for each of the three stakeholder assessments as well as examining specific elements independently.

**Figure 4:**
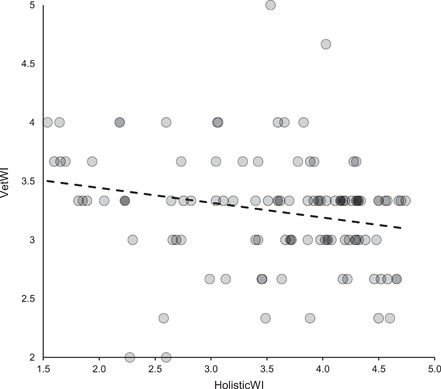
Relationship between the VetWI and the HolisticWI (Spearman's correlation, *r*_s_ (107)= −.27514, *p* (two‐tailed) = .00413)

**Figure 5:**
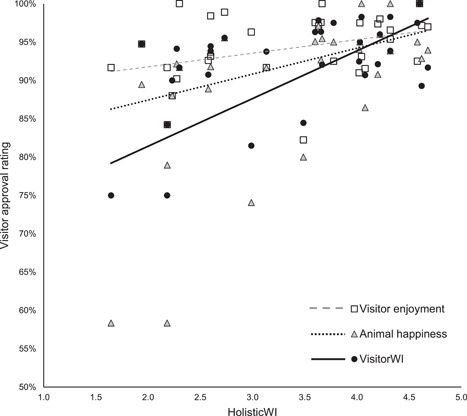
The relationship between HolisticWI and VisitorWI (Spearman's correlation, *r*_s_ (32) = .45254, *p* (two‐tailed) = .00931), the visitors' approval rating of the happiness of resident animals in each habitat (Spearman's correlation, *r*_s_ (32) = .63474, *p* (two‐tailed) = .0001) and the visitors' enjoyment approval rating of each habitat (Spearman's correlation, *r*_s_ (32) = .37468, *p* (two‐tailed) = .03462)

**Figure 6:**
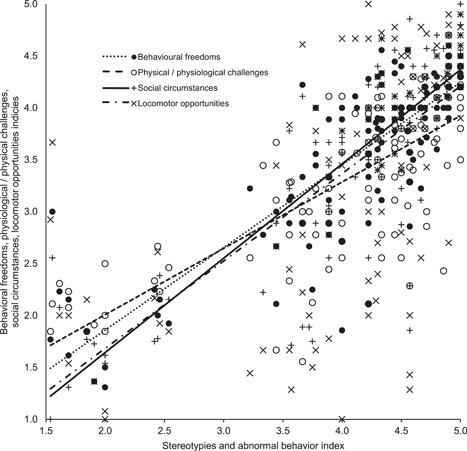
The relationship between animal carers perceived level of stereotypies and/or abnormal behaviours within each habitat and their assessments of the extent to which habitats curtailed behavioural freedoms (Spearman's correlation, *r*_s_ (130) = .79575, *p* (two‐tailed) = .000), predisposed resident animals to physiological and or physical challenges (Spearman's correlation, *r*_s_ (130) = .74258, *p* (two‐tailed) = .000), failed to cater for resident animal's social requirements (Spearman's correlation, *r*_s_ (130) = .83431, *p* (two‐tailed) = .000), and curtailed their locomotor opportunities (Spearman's correlation, *r*_s_ (130) *r*_s_ = .67523, *p* (two‐tailed) = .000)

The HolisticWI was the most inclusive and broad‐based of the three assessments comprising elements relating to health/biological functioning and natural living, together with insights into the affective states of resident animals by considering the prevalence of stereotypic and other abnormal behaviors (Fraser, 2008a; Webb et al., 2019; Weary & Robbins, 2019) and as such serves as the reference point for the more limited veterinary and visitor‐based conceptions of welfare. The audit revealed there was a significant negative correlation between the HolisticWI and the VetWI (Spearman's correlation, *r*_s_ (107) = −.27514, *p* (two‐tailed) = .00413, see Figure 4). There was, however, a positive correlation between the HolisticWI and the VisitorWI (Spearman's correlation, *r*_s_ (32) = .45254, *p* (two‐tailed) = .00931), the visitors' approval rating of the happiness of resident animals in each habitat (Spearman's correlation, *r*_s_ (32) = .63474, *p* (two‐tailed) = .0001) and the visitors' enjoyment approval rating of each habitat (Spearman's correlation, *r*_s_ (32) = .37468, *p* (two‐tailed) = .03462, see Figure 5). There was no significant correlation between HolisticWI and the visitors' health‐based approval rating of each habitat (Spearman's correlation, *r*_s_ (32) = .14445, *p* (two‐tailed) = .43026). Among the criteria within the HolisticWI, the animal carers' perceived prevalence of stereotypies and/or abnormal behaviours within each habitat, significantly correlated with their assessments of the extent to which habitats failed to cater for resident animal's social requirements (Spearman's correlation, *r*_s_ (130) = .83431, *p* (two‐tailed) = .000), curtailed behavioural freedoms (Spearman's correlation, *r*_s_ (130) = .79575, *p* (two‐tailed) = .000), predisposed resident animals to physiological and or physical challenges (Spearman's correlation, *r*_s_ (130)= .74258, *p* (two‐tailed) = .000), and curtailed their locomotor opportunities (Spearman's correlation, *r*_s_ (130) = .67523, *p* (two‐tailed) = .000, see Figure 6).

**Figure 7:**
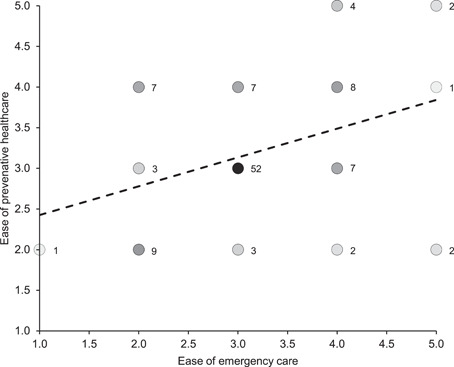
The relationship between ease of emergency care correlated with ease of preventative care. The frequency of corresponding habitats is represented numerically and by the darkness of circles (Spearman's correlation, *r*_s_ (107) = .32497, *p* (two‐tailed) = .00064)

**Figure 8:**
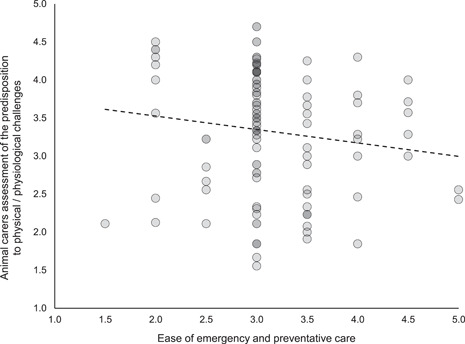
The relationship between ease of emergency and preventative care correlated with animal carers assessment of the predisposition of animals to physical and physiological challenges (Spearman's correlation, *r*_s_ (107) = −.19513, *p* (two‐tailed) = .04299)

As veterinary staff are highly influential in matters pertaining to animal welfare within zoos and aquariums but inevitably have a health‐centred focus, it is important to understand how the animal welfare conceptions of this stakeholder group related to that of the welfare conceptions/perceptions of visitors, and other more holistic animal welfare conceptions such as the one set out here. In addition to the inverse correlation with the HolisticWI (see Figure 4), there was found to be no significant correlation between the VetWI and the VisitorWI (Spearman's correlation, *r*_s_ (27) = −.00682, *p* (two‐tailed) = .97307), the visitors' perception of animal health (Spearman's correlation, *r*_s_ (27) = .01739, *p* (two‐tailed) = .93138) nor the extent to which animal carers' believed habitats predisposed resident animals to physiological and/or physical challenges (Spearman's correlation, *r*_s_ (129) = −.1683, *p* (two‐tailed) = .08167). Ease of emergency care correlated with ease of preventative care (Spearman's correlation, *r*_s_ (107) = .32497, *p* (two‐tailed) = .00064, see Figure 7), however, the veterinary team's assessment of morbidity and mortality did not correlate with the veterinary team's combined assessment of the ease of emergency and preventative care (Spearman's correlation, *r*_s_ (107) = −.0864, *p* (two‐tailed) = .37392), nor with ease of emergency care (Spearman's correlation, *r*_s_ (107) = −.06211, *p* (two‐tailed) = .52308) or preventative care independently (Spearman's correlation, *r*_s_ (107 = −.08311, *p* (two‐tailed) = .39248). The veterinary team's assessment of morbidity and mortality did not correlate with the animal carers' assessment of the predisposition of animals to physical and physiological challenges (Spearman's correlation, *r*_s_ (107) = −.01498, *p* (two‐tailed) = .87774), but the veterinary team's combined assessment of the ease of emergency and preventative care inversely correlated with the animal carers' assessment of the predisposition of animals to physical and physiological challenges (Spearman's correlation, *r*_s_ (107) = −.19513, *p* (two‐tailed) = .04299, see Figure 8).

**Figure 9:**
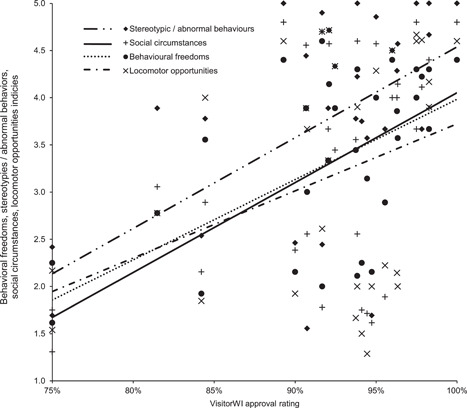
The relationship between VisitorWI and the animal carers' assessment of behavioural freedoms (Spearman's correlation, *r*_s_ (32) = .50018, *p* (two‐tailed) = .00355), the extent to which habitats catered for the social requirements of resident animals (Spearman's correlation, *r*_s_ (32) = .3996, *p* (two‐tailed) = .02346), and the infrequency with which animal careers perceived animals expressed stereotypies and/or abnormal behaviours within each habitat (Spearman's correlation, *r*_s_ (32) = .46791, *p* (two‐tailed) = .00692)

**Figure 10:**
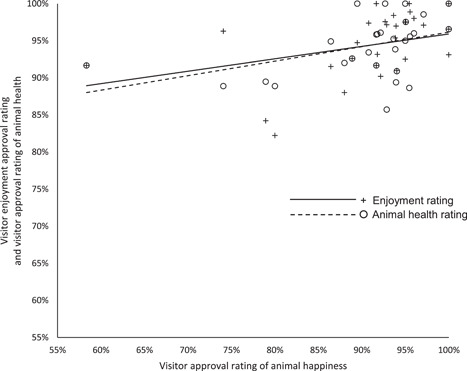
The relationship between visitors' perceptions of animal happiness and the visitors' enjoyment of that habitat (Spearman's correlation, *r*_s_ (29) = .55462, *p* (two‐tailed) = .00179) and their approval rating of the health of animals (Spearman's correlation, *r*_s_ (31) = .51306, *p* (two‐tailed) = .00268)

Finally, the visitors' assessments enable us to understand how ‘folk' conceptions of health and happiness relate to more evidenced‐based conceptions of animal welfare and management as well as the visitors' experiences of habitats. While VisitorWI correlated with the HolisticWI (see Figure 5) but not with the VetWI, there was also a significant correlation between the VisitorWI and the animal carers' assessment of behavioral freedoms (Spearman's correlation, *r*_s_ (32) =  .50018, *p* (two‐tailed) = .00355), the infrequency with which animal carers perceived animals expressed stereotypies and/or abnormal behaviours within each habitat (Spearman's correlation, *r*_s_ (32) = .46791, *p* (two‐tailed) = .00692) and the extent to which animal carers perceived habitats to cater for the social requirements of resident animals (Spearman's correlation, *r*_s_ (32) = .3996, *p* (two‐tailed) = .02346, see Figure 9). There was, however, no significant correlation between VisitorWI and the extent to which animal carers considered habitats predisposed resident animals to physiological and/or physical challenges (Spearman's correlation, *r*_s_ (32) = .30097, *p* (two‐tailed) = .09415) or curtailed the locomotor opportunities of a habitat's resident animals (Spearman's correlation, *r*_s_ (32) = .32036, *p* (two‐tailed) = .07384). There was a significant correlation between the visitors' approval rating of the happiness of animals with their approval rating of their health (Spearman's correlation, *r*_s_ (31) = .51306, *p* (two‐tailed) = .00268) and the extent to which visitors enjoyed habitats (Spearman's correlation, *r*_s_ (29) = .55462, *p* (two‐tailed) = .00179, see Figure 10). Visitor perceptions of animal health did not however correlate with enjoyment of habitats by visitors (Spearman's correlation, *r*_s_ (29) = .18065, *p* (two‐tailed) = .34835).

## 4 DISCUSSION

Since the audit was undertaken primarily to inform institutional animal welfare strategies covering aspects as diverse as daily animal care operations through to longer term collection and facility master‐planning, there are some constraints that need to be considered. As all habitats in this large and diverse wildlife facility needed to be assessed within a limited timeframe, the methodology employed is broader in its approach than one that might be undertaken for a single species, individual animal, or even for a single habitat. Furthermore, while mixed species communities that made up most habitats in this facility have the potential to enhance the welfare of captive animals (see Veasey & Hammer, 2012), they also make assessing individual animal welfare and auditing facility‐based animal welfare performance more challenging. Finally, because the veterinary team's data was provided as a consensus view rather than as independent assessments from each team member, that data must be treated with some degree of caution, however, it is nonetheless an important data set since it reflects the collective, if non‐independent view of a veterinary team on what they believe was important to safeguard welfare using criteria of their choosing across many habitats.

Despite these potential constraints, evidently, such a comprehensive facility‐wide audit encompassing three distinct perspectives of animal welfare  reveals valuable insights that would be unobtainable from more in‐depth assessments across a smaller number of habitats or individual animals which not only have relevance to the institution concerned, but also more generally.

### 4.1 Differing welfare conceptions

**Figure 11:**
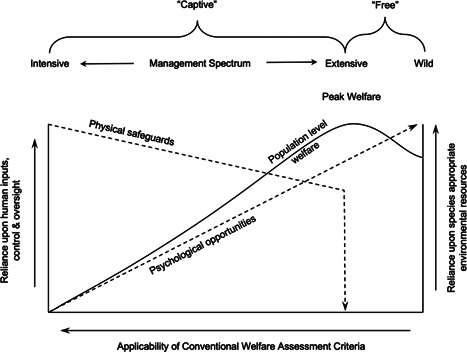
Schematic representation of the relationship between efforts to safeguard physical health and the availability of psychological opportunities for managed animals across the spectrum of intensive and extensive management systems and how this might impact population‐level animal welfare (modified from Veasey, 2017)

The audit demonstrates how welfare prioritisation based on differing conceptions of animal welfare could conflict with each other, and ultimatately with welfare states as experienced by individual animals as represented here by the HolisticWI. The inverse relationship between the VetWI and the HolisticWI (see Figure 4) clearly evidences this and supports the proposition that there is a tension between physical and psychological priorities in captive animal welfare provisioning (2017). Veasey (2017) reasoned that smaller, simpler, more intensively managed environments can facilitate oversight and the capacity to intervene; criteria reflected in the VetWI, but suggested larger, more species appropriately complex habitats in which intensive management and oversight are to some extent inherently impeded, typically cater better for the psychological needs of animals; which are better reflected in the criteria used in the HolisticWI (see Figure 11). It should be noted that neither the VetWI nor the HolisticWI are measures of animal welfare *per se*, rather they are attempts to audit aspects of a habitat and its associated management systems to cater for animal welfare. The veterinary team's self‐selected criteria inevitably reflects their roles and priorities, whereas the animal care team were provided training and calibrated assessment criteria by the author that were intentionally broad‐based and encompassing three principal animal welfare constructs. And so, these results do not necessarily suggest that the veterinary and animal care teams disagree over the welfare status of individual animals in this facility, rather they suggest that what is considered desirable by veterinary staff to manage animal welfare, may not align with the criteria necessary to manage animal welfare as viewed from a more holistic perspective such as that set out here by the author. This highlights the necessity of being mindful of differing animal welfare conceptions when assessing animal welfare performance, developing animal welfare strategies or designing habitats, and also the need to optimise healthcare provisioning rather than maximising it in pursuit of peak animal welfare (Veasey 2017).

While the extent to which animal carers perceived animals expressed abnormal behaviors including stereotypies were shown to be significantly different from all other criteria making up the HolsticWI (see Figure 1), it is notable this criterion correlated with all other criteria making up the HolisticWI (see Figure 6). While it is not possible to definitively establish a causal relationship between the curtailment of appropriate social, behavioral and locomotor opportunities and the extent to which animals were perceived to stereotype or express abnormal behaviors, this would appear to be the most logical explanation. Arguably, further weight is lent to this hypothesis by the significant relationship between VisitorWI and the curtailment of appropriate social and behavioral opportunities and the extent to which animals were perceived to stereotype or express abnormal behaviors (see Figure 9). The correlation between abnormal behaviors including stereotypies and physical challenges is perhaps more likely attributable to both being the result of compromised welfare which subsequently covary, rather than a causal relationship between the two. Nonetheless, these findings add further credence to the value of assessing the extent to which animals express abnormal behaviors including stereotypies as a proxy welfare indicator in the absence of more comprehensive, broad‐based data.

The ease with which the veterinary team considered emergency care could be provisioned perhaps unsurprisingly correlated strongly with their perception of the ease of provisioning of preventative care (see Figure 7) which conforms with Veasey's observations on the relationship between scale, environmental and social complexity and medical oversight and management ease (2017, see Figure 11). However, it is interesting that neither the ease of the provision of preventative care and the ease of the provision of emergency care independently or combined, correlated with the veterinary team's assessment of the predisposition of resident animals to morbidity and mortality, nor the animal carers' assessment of the extent to which habitats predisposed resident animals to physiological/physical challenges. Notwithstanding the limitations of the veterinary data set, there are several possible explanations for these findings. It could be that the extent to which the veterinary team felt they could provide emergency and preventative healthcare had no impact on overall health outcomes, however, it is also possible that species that are unduly predisposed to health challenges under prevailing management and habitat paradigms, may in fact be afforded greater opportunities for medical interventions through management or facility design, which might still be insufficient to overcome their intrinsic tendency to health challenges. Furthermore, and perhaps most likely, it is possible that while complex, naturalistic environments may hinder medical management and oversight, they may also predispose animals to better physical health as well as enhancing their psychological health (Ross & Mason 2017). Regardless of the potential causal relationships, the audit illustrates that the capacity for medical oversight and intervention does not necessarily correlate with health or welfare.

That the animal carers' assessment of the extent to which habitats predisposed resident animals to physiological/physical challenges did not correlate with the veterinary team's assessment of morbidity and mortality is potentially the result of differing perceptions of the true situation or a consequence of the difference between cause and effect; the animal carers' assessment considers the extent to which an environment predisposes animals to physical challenges emphasising causality, whereas the veterinary assessment considers outcomes in the form of morbidity and mortality. And so, while some habitats might theoretically predispose animals to physical challenges more than others, efforts undertaken to mitigate those effects may mean it need not necessarily follow that they result in increased impacts on morbidity and mortality.

That the VetWI did not correlate with the visitors' perception of animal health nor the VisitorWI is at first not surprising since the former is a data set provided by a highly experienced and qualified team of experts reflecting their collective view of what is considered to be important to safeguard physical wellbeing, whereas, at best, the visitors' perception of animal health and VisitorWI arguably represents a ‘folk' perception of health and happiness (see Webb et al., 2019). However, the fact that both the VisitorWI and visitors' perception of animal happiness correlated with the HolisticWI is a genuinely fascinating result (see Figure 5). This is particularly so considering the VisitorWI also correlated with the extent to which habitats catered for the social, behavioural and locomotor opportunities for resident animals as well as the extent to which animals were perceived to be free from stereotypic or abnormal behaviours by the animal care team (see Figure 9). It is not possible to determine whether the correlation between visitor perceptions of animal happiness and health and the extent to which habitats catered for the social, behavioural and locomotor opportunities and the freedom from abnormal behaviours including stereotypies was a result of visitors picking up on the environmental drivers of poor welfare which appear to manifest in the presence or absence of stereotypic or abnormal behaviours, or whether they are picking up on the presence or absence of the abnormal behaviours themselves. Nonetheless, the strength of the correlation between HolisticWI and VisitorWI, visitor perceptions of animal happiness alone and their enjoyment of habitats (see Figure 5) suggests that visitors of this wildlife attraction possess an understanding of animal happiness and health that coincides with holistic conceptions of animal welfare as set out in this audit process, and that it is a key driver in their enjoyment of each habitat.

### 4.2 The case for an animal welfare consensus based around the affective states of animals

Veasey previously argued that animal welfare conceptions and strategies frequently reflected how welfare was measured, typically emphasising the most tangible health and welfare metrics, rather than how welfare is experienced by the animals, and that this approach was ultimately detrimental to animal welfare (2017). This audit clearly illustrates the potential conflict that could arise if, for example, veterinary derived welfare assessment criteria were used in isolation to determine welfare strategies or facility design. However, the failure to fully comprehend the difference between how welfare is measured and how it is experienced by animals is not restricted to welfare management but is also manifest in how many wildlife attractions engage with the public on animal welfare issues. The nuanced nature of animal welfare and the lack of consensus on how animal welfare is defined creates space for animal welfare controversies to be debated around tangible metrics relating to physical health and longevity, frequently combined with arguably irrelevant consequentialist arguments referencing educational or conservation benefits beyond the animals for which welfare concern is being discussed (see CNN, 2013; SeaWorld, 2021; Wood 2010). However, it appears increasingly apparent to those with an interest in animal welfare, that it is possible for an animal to have a long, healthy but ultimately unhappy life, and that the mental state of individual animals, should not be subordinate to their health. The fact that visitors ranked animal health higher than they did animal happiness in their approval rating (see Figure 3) and yet it was animal happiness that correlated marginally more strongly with their enjoyment of those habitats than health (see Figure 10) could lend further support to this proposition. Furthermore, the reliance on consequentialist defenses to welfare concerns relating to the benefits to wildlife generally, the species concerned or society, are arguably as likely to reinforce concerns over individual animal welfare as they are to justify welfare failures, and ultimately, such arguments are irrelevant to the welfare of the individual animals concerned.

And so, the lack of consensus on how animal welfare is defined arguably presents as much of a challenge to those opposed to zoos and aquariums on animal welfare grounds, as it does to those responsible for managing welfare within zoos and aquariums. However, the fact that visitors to this wildlife attraction appear to have an intuitive understanding of good animal welfare/animal happiness and that this influences their experiences suggests that ensuring the psychological needs of animals are met, is necessary to assuage public concerns over animal welfare; an issue that is increasingly important to them.

The contrasting histories of the social acceptability of captive polar bears (*Ursus maritimus*) in the British Isles and captive cetaceans around the world appear to support this proposition. Despite growing public concern for captive polar bear welfare and the publication of three critical reports on the welfare of British and Irish polar bears during the 1980s and 1990s (Ames, 1993; Horsmann, 1986; Ormrod, 1992), the recent revival of the keeping polar bears in UK wildlife parks and the social acceptability that underpins that, is no doubt a result of the acceptance and subsequent implementation of the recommendations of those reports, tangibly manifest in the twentyfold increase in average polar bear habitat size that has occurred within the United Kingdom during the 2000s. By way of contrast, the collapse in the social acceptability of keeping cetaceans in a variety of jurisdictions around the world during the 2010s (Scott‐Reid, 2019), is arguably a consequence of a more defensive response by holders against a backdrop of growing public concerns, likely exacerbated by the application of imperfect and inconsistent animal welfare definitions that fail to effectively prioritise the affective states of animals and address the fundamental concerns of the public. Even Kagan et al. (2015) when ambitiously proposing a universal framework for animal welfare in zoos failed to provide a definition to which they subscribed.

To diminish this ambiguity, which has been identified as a source of public concern and skepticism in respect of animal based attractions (Marinova & Fox, 2019), it is likely useful to consider animal welfare through the lens of happiness (see Broom, 2007; Veasey 2017; Webb et al., 2019). Many will claim that to do so is anthropomorphic, counterproductive, and even dangerous (see J. D. Rose, 2007; Tannenbaum, 2002) and while there are of course legitimate concerns in assuming that which makes a human happy is the same as that which makes an animal happy, it does not follow that happiness is unique to humans. Indeed, it is probable that most vertebrates can experience happiness, as they can experience fear, anger or despair since these, and other affective states have an adaptive value (Greiveldinger, 2009; Yeates, 2010). Furthermore, subjective assessments of individual animal happiness have been shown to correlate with broad‐based assessments of animal welfare in this audit and other studies (see Robinson et al., 2016; 2021). And so, the use of the term happiness in relation to animal welfare should not necessarily be considered as anthropomorphic or unscientific *per se*, rather attributing human‐like drivers for happiness/positive animal welfare would be if they were not based upon sound reasoning or a solid evidential base. The benefit of applying such a filter to animal welfare is it better reflects the growing concerns of many western societies and makes it impossible to disregard the affective states of animals, which are currently all too frequently subservient to the more quantifiable components of physical wellbeing (Veasey, 2017). To keep pace with societal concerns relating to animal welfare, zoos and aquariums should, therefore, consider conceptions of animal welfare that consider poor welfare to occur when animals experience severe or chronic states of mental suffering (unhappiness, distress, depression, pain, anxiety, hunger, thirst etc.), and good welfare to occur when animals experience positive emotional states (happiness, enjoyment/pleasure, satiation, excitement, contentment, relaxation etc.), and negligible mental suffering (see Mason & Veasey, 2009a, 2009b, 2010).

Such a conception subscribes to the belief that animal welfare is all to do with the feelings of animals (see Duncan & Petherick, 1991; Duncan, 2002; Veasey, 2017), physical health and natural living are excluded from this definition because while they can readily impact animal welfare (as this audit appears to confirm), they do so, only when they impact the feelings of animals. Moreover, physical health and natural living are two of many factors that can impact animal welfare such as an animal's thermal, social and physical environment and so on. And so, not only is the inclusion of health and natural living redundant in defining welfare (if not in influencing and assessing welfare as evidenced here), their inclusion can be problematic since the pursuit of good health and replicating the naturalness of an animal's life, including its many natural stressors can be detrimental to an animal's welfare (Veasey, 2017; Veasey et al., 1996a, 1996b). And so, while they are important considerations (as are many others) in establishing animal welfare priorities and in assessing welfare, they should be considered in context as means to achieve good welfare rather than defining goals in of themselves.

### 4.3 Animal welfare as an opportunity rather than a cost

In addition to illustrating the value in establishing consensus on a definition of animal welfare that prioritizes the affective states of animals, the strong correlation between visitors' perceptions of animal happiness, the HolisticWI and visitors' enjoyment of those habitats (see Figures 5 and 10) suggests the quality of an animal's life within a wildlife attraction directly influences the visitors' experiences of a wildlife attraction (Veasey 2005, 2019) and supports the findings of Melfi & Mccormick (2004) which found visitor preferences for primate enclosures covaried with welfare. This challenges the widely expressed belief that animal welfare and commercial priorities are inherently in conflict in zoos and aquariums (see Born Free, 2021; Keulartz, 2015; Morino, 2017; PETA, 2021; N. A. Rose & Parsons, 2019; Wolfensohn et al., 2018). A belief in such a conflict appears to increasingly be reflected in legislation; in Canada, the Ending the Captivity of Whales and Dolphins Act (2019) states an offence is committed when 'captive cetaceans are used for performance for entertainment purposes' (Parliament of Canada, 2019) and similarly, the proposed 'Jane Goodall Act' as set out in its first reading in the Canadian Parliament expressly prohibits 'captivity of certain animals for entertainment purposes' (Parliament of Canada, 2020), and yet both Acts permit captivity for such species where 'entertainment' is not occurring as if to suggest that public entertainment is in some way relevant to the welfare of the animals concerned. While it may often be the case that animal welfare can be compromised in the pursuit of entertaining humans, it does not follow that this is inevitably so, nor does it follow that if people are entertained by animals, the welfare of those animals is subsequently compromised as the findings of this audit suggest.

Furthermore, the commercial impacts of welfare controversies on a wide array of wildlife attractions has been shown to be devastating, (see, e.g., CBC, 2016; Kerr, 2019; Neate, 2015) suggesting that the commercial viability of zoos and aquariums is ultimately dependent on welfare, not in competition with it. And so, while the positioning of animal welfare as a moral obligation is arguably a defining characteristic of a ‘good' zoo or aquarium, it is not a priority that should be viewed as competing with financial objectives. In fact, the long‐term commercial viability of wildlife attractions in many ‘western' countries at least, is likely to be dependent on the ability of facilities to demonstrably deliver high standards of animal welfare because it shapes the visitor experience, and insulates institutions against costly public relations catastrophes.

While improvements in animal welfare provisioning over the last half‐century within most accredited ‘western' zoos, and likely many more besides would be hard to dispute (see Kitchener & MacDonald, 2002; Finch et al., 2020; Tidière et al., 2016), these improvements have likely at best, kept pace with a growing awareness of, and concern for animal welfare among the general populace (see Marinova & Fox, 2019; Robbins et al., 2018; L. E. Webb et al., 2019). Furthermore, it is also likely that zoos and aquariums have made more fundamental improvements in the provisioning of physical wellbeing than they have in delivering psychological wellbeing (Veasey, 2017) with most species now living longer in zoos than they would in the wild (see Tidière et al., 2016), but with stereotypies and other abnormal behaviours still being routine for many species (see Clubb & Mason, 2003, 2007; Mason & Veasey, 2009a, 2009b, 2010; Swaisgood & Shepherdson, 2005). While it is difficult to determine how much of this can be attributed to the prevalence of health‐centred animal welfare conceptions in shaping welfare management, there is evidently still a need for the gains that have been made regarding physical wellbeing to be replicated in improvements in psychological wellbeing in zoos and aquariums.

As this audit is representative of a single large North American wildlife attraction, care must be taken extrapolating findings relating to the welfare conceptions of visitors and the veterinary teams to different locations. However, the HolisticWI is intended to reflect the needs of the animals themselves independent of any non‐scientific conception of welfare or cultural emphasis and so is more universally applicable. Further research exploring regional variations in stakeholder conceptions of welfare benchmarked against the HolisticWI is clearly warranted. Notwithstanding the potential cultural specificity of these findings, the audit suggest visitors to North American zoos and aquariums are probably more cognizant of animal welfare than they have been given credit for, and that they are potentially more sensitive to the affective states of animals than they are to the health status of animals on display. And so, the failure to meet the psychological needs of animals should be considered as an existential threat to the sector, and to address this moral and strategic priority, zoos and aquariums are encouraged to assess the welfare status of their facilities and animals with a particular emphasis on the affective states of animals as reflected in the HolisticWI, and subsequently develop ongoing strategies to address those needs.

## Data Availability

Due to commitments to institutional anonymity, data is not publicly available.
